# Output power-density limit of a thermoradiative diode with an intermediate band

**DOI:** 10.1038/s41598-025-91800-8

**Published:** 2025-03-03

**Authors:** Yukihiro Harada, Fuka Nishii, Takashi Kita

**Affiliations:** 1 Department of Electrical and Electronic Engineering, Graduate School of Engineering, Kobe University, 1-1 Rokkodai, Nada, Kobe, 657-8501 Japan; 2 Department of Electrical and Electronic Engineering, Faculty of Engineering, Kobe University, 1-1 Rokkodai, Nada, Kobe, 657-8501 Japan

**Keywords:** Energy harvesting, Electrical and electronic engineering, Photonic devices

## Abstract

The application of the thermoradiative effect of photodiodes, in which photons are emitted to a cold reservoir in the far-field, is a promising approach for renewable electricity generation. Here we derive the radiative limit of the output power density of an ideal thermoradiative diode (TRD) with an intermediate band (IB) using detailed balance calculations. The output power density of an ideal IB-TRD with a given bandgap energy and an optimal IB position increases with the device temperature, and simultaneously the optimal position of the IB shifts away from the mid-gap position due to the current matching constraint. Since the intrinsic carrier density needs to be significantly lower than the doping concentration to form a *p*–*n* junction at the operating temperature, IB-TRDs can be advantageous compared to single-junction TRDs consisting of narrow-bandgap semiconductors.

## Introduction

Thermoradiative power generation has attracted attention as a process where the emission of infrared photons can be used to generate electricity^1–3^. So-called thermoradiative diodes (TRDs) operate under negative illumination conditions, that is, the density of photons emitted by a TRD is larger than that absorbed by the TRD (the device temperature is higher than that of the environment)^4^. The efficiency limit of thermoradiative energy conversion by far-field radiative energy transfer has been discussed using detailed balance calculations^5,6^ and thermodynamics^6^; the energy conversion efficiency approaches the Carnot limit in TRDs consisting of wide-bandgap semiconductors. However, there is a trade-off between the efficiency and the output power density, because the output power density approaches zero as the bandgap energy becomes larger. Because the output power density of a TRD increases as the bandgap becomes narrower, near-room-temperature thermoradiative power generation has been demonstrated using HgCdTe^4,7,8^ and InAsSb^9^, which is used in commercially available infrared photodetectors.

Another theoretical result is that the output power density of a TRD increases with the device temperature^5,6^. A theoretical work that takes optical losses, sub-bandgap radiation and non-radiative losses into account predicted that a thin-film InSb-based thermoradiative system at 500 K can achieve an output power density of 113 W/m$^2$ if the environmental temperature is 300 K^10^. On the other hand, the formation of an InSb-based *p–n* junction is difficult because the intrinsic carrier density of InSb exceeds 10$^{17} \mathrm{~cm}^{-3}$ at 500 K^11^ (considering a bandgap energy of 0.09 eV^10^). The high-temperature operation of single-junction (SJ) TRDs consisting of narrow-bandgap semiconductors is thus challenging.

On the other hand, various intermediate-band (IB) solar cell architectures have been proposed to realize conversion efficiencies that exceed the conversion efficiency limit for SJ solar cells^12–15^. The key idea of the IB solar cell architecture is to enable the use of below-bandgap photons by two-step photon absorption via an IB placed in the bandgap of the host semiconductor. Two-step photon absorption via an IB has been demonstrated in IB solar cells based on quantum dots^16–19^, highly mismatched alloys^20^ and heterostructures^21,22^. In this work, we focus on the radiative transitions via IBs for thermoradiative power generation, because the bandgaps that correspond to these transitions are narrow while the bandgap of the host semiconductor is relatively wide. So far, output power density and efficiency of an ideal TRD with an IB (IB-TRD) has been theoretically investigated using the detailed balance approach with narrow bandgap materials ($E_\textrm{g}<$ 0.32 eV)^23–25^. Besides, it is assumed that the absorptivity for each transition has no overlap between the absorption coefficients^23–26^, resulting in the degraded performance of the IB-TRD^27^. In this work, we studied the output power density of IB-TRDs with wider band-gap materials taking into account the overlap between the absorption coefficients and the intrinsic carrier concentration constraint. The overlap between the absorption coefficients results in the superior performance of the IB-TRD. Our results indicate that IB-TRDs can be useful for applications where operation under both positive and negative illumination conditions is required.

## Methods

We performed the calculations using the Julia programming language^28^.

### Detailed balance analysis of current-density–voltage relation

**Fig. 1:**
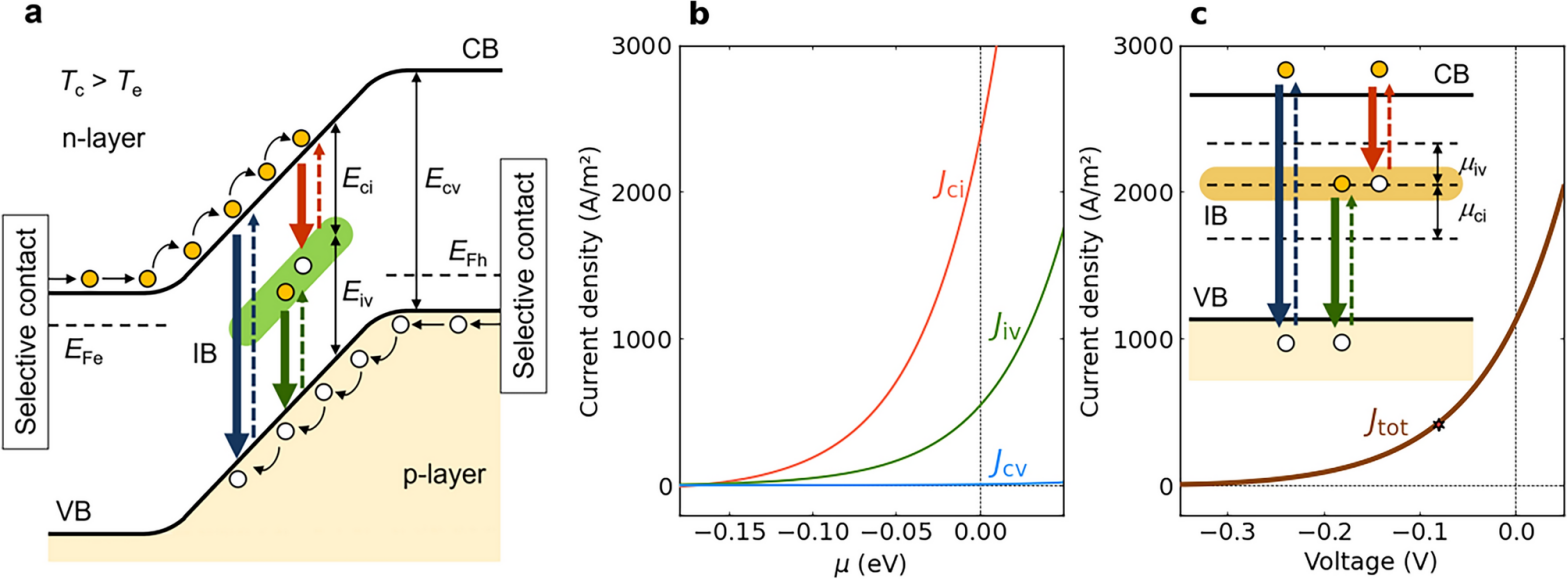
Energy band diagram and current-density–voltage characteristics of an ideal IB-TRD. **a** Schematic energy band diagram of an IB-TRD during operation. The device operates under negative illumination conditions; the temperature of the device, $T_\textrm{c}$, is higher than that of the environment, $T_\textrm{e}$, and therefore the photon flux exceeds that of the absorbed photons. The orange and white circles represent electrons and holes, respectively. The difference between the quasi-Fermi levels of the electrons and holes ($E_\textrm{Fe}$ and $E_\textrm{Fh}$, respectively), determines the output voltage. The conduction and the valence bands are denoted by CB and VB, respectively. **b** Current-density-chemical-potential characteristics of an ideal IB-TRD with $E_\textrm{cv}$ = 0.5 eV and $E_\textrm{ci}$ = 0.22 eV for $T_\textrm{c}$ = 500 K and $T_\textrm{e}$ = 300 K. $J_\textrm{cv}$, $J_\textrm{ci}$ and $J_\textrm{iv}$ are the current densities created by the CB–VB-, CB–IB- and IB–VB-transitions, respectively. **c** Current-density–voltage characteristics of an ideal IB-TRD in **b**. $J_\textrm{tot}$ is the total current density. The star represents the maximum power point. The inset shows the energy band diagram for the detailed balance calculation. $\mu _\textrm{ci}$ and $\mu _\textrm{iv}$ are the chemical potentials for the CB–IB- and IB–VB-transitions, respectively.

To explain the basic properties of an IB-TRD, we show a schematic energy band diagram in Fig. 1a. We assume that a semiconductor having the IB in the bandgap is situated between the *n*- and *p*-emitters as is the case with the IB solar cells^29^. The orange and white circles represent electrons and holes, respectively. A TRD operates under negative illumination conditions, that is, the temperature of the device, $T_\textrm{c}$, is higher than that of the environment, $T_\textrm{e}$. The black arrows illustrate how thermalised electrons in the conduction band (CB) of the n-layer and holes in the valence band (VB) of the p-layer diffuse towards the space-charge region at the centre. The blue, red and green downward arrows represent radiative recombination via band-to-band transitions in the host semiconductor (bandgap energy: $E_\textrm{cv}$)^10^ and the transitions involving the IB (bandgap energies: $E_\textrm{ci}$ and $E_\textrm{iv}$), while the upward arrows represent excitation by the infrared radiation from the environmental surroundings. These recombination processes induce a negative open-circuit voltage, which means that the electron quasi-Fermi level $E_\textrm{Fe}$ is below the hole quasi-Fermi level $E_\textrm{Fh}$. Furthermore, the total quasi-Fermi level splitting needs to be equal to the sum of the sub-bandgap quasi-Fermi level splittings (like in the case of IB solar cells^12^). For the calculations in this work, we assumed that the energy width of the IB is zero, which means $E_\textrm{cv} = E_\textrm{ci} + E_\textrm{iv}$. When an external load is connected to this device, the recombination-induced reduction in the density of electrons in the CB is partially compensated by a current through the external circuit, which equals the hole current flow to the VB, and this mechanism ensures a continuous charge flow through the TRD^10^. Compared to a TRD without an IB (hereafter referred to as an SJ-TRD), the two-step transitions via the IB create an additional current flow in the IB-TRD.

The output power density of a TRD can be calculated by the detailed balance analysis of the current-density–voltage relation^5,6^. To determine the output power density, we assumed that the absorptivity for each of the three possible types of transitions (CB–VB, CB–IB and IB–VB) is constant for photon energies larger than the corresponding bandgap energy, and that the absorption coefficients of the three transition channels overlap. In general, an overlap between the absorption coefficients reduces the efficiency of an IB solar cell^30^, but an overlap between the transitions via the IB can also improve the efficiency in the case of low to moderate concentration ratios^31^. The emitted photon flux density *N* in a given photon energy range defined by $E_\textrm{min}$ and $E_\textrm{max}$ can be calculated using the generalized Planck law, which includes the effect of the chemical potential $\mu$^32^:

1 $$\begin{aligned} N(E_\textrm{min},E_\textrm{max}, T, \mu , a) = \frac{2 \pi }{h^3 c^2} \int _{E_\textrm{min}}^{E_\textrm{max}} \frac{a(E) E^2}{\exp \left( \frac{E - \mu }{k_\textrm{B} T} \right) -1} \textrm{d}E \,\,\, , \end{aligned}$$

where *T* is the temperature, *h* is Planck’s constant, *c* is the speed of light in a vacuum, $k_\textrm{B}$ is Boltzmann’s constant and *a* is the absorptivity as function of the photon energy *E*, which is equal to the emissivity according to Kirchhoff’s law. In this work, we assumed that the absorptivity $a_i$ of each of the three possible types of transitions $\; (i \in \textrm{ci, iv, cv} )$ is constant for photon energies larger than the corresponding bandgap energy $E_i$, and the solid angles of absorption and emission are $\pi$ by a perfect mirror at the back side of the TRD.

The current densities in the radiative limit are given by the following equation:

2 $$\begin{aligned} \frac{J_i}{q} = N(E_i, \infty , T_\textrm{c}, \mu _i, a_i^{*}) - N(E_i, \infty , T_\textrm{e}, 0, a_i^{*}) \,\,\, , \end{aligned}$$

where $\mu _i$ is the quasi-Fermi level splitting for each transition channel, *q* is the elementary charge, $T_\textrm{c}$ is the temperature of the device, $T_\textrm{e}$ (= 300 K) is the temperature of the environment and $a_i^{*}$ is an effective absorptivity that accounts for the overlap between the absorption coefficients. $a_i^{*}$ is defined as

3 $$\begin{aligned} a_i^{*} (E) = \left\{ 1 - \prod _{j} \left[ 1 - a_j (E) \right] \right\} \frac{a_i (E)}{\sum _{j} a_j (E)} \,\,\, . \end{aligned}$$

The term $\prod _{j} \left[ 1 - a_j (E) \right]$ describes the ratio that cannot be absorbed by the three transition channels, which is needed when the absorptivity of all transition channels is less than unity. Therefore, $1 - \prod _{j} \left[ 1 - a_j (E) \right]$ describes the total absorptivity at the energy *E* by the three transition channels. The latter term describes the ratio of the effective absorptivity for each transition channel. In this work, the absorptivity $a_i$ is assumed to be unity above the bandgap energy if the value is not described. It has been reported that the optimal device thickness exists with fixed absorption coefficients in the IB solar cells by taking into account the reabsorption process^30^. Here, we use the absorptivity as a variable instead of the device thickness, which is determined by the device thickness and absorption coefficient. Besides, near-field radiative transfer can improve the maximum work flux of the TRD^34,35^. The radiation control based on the Purcell effect will further improve the device performance.

The total current density $J_\textrm{tot}$ can be obtained from the following Eq. 4, which uses the current matching constraint for the transitions via the IB, and the output voltage *V* is given by Eq. 5, which uses the fact that the total quasi-Fermi level splitting needs to be equal to the sum of the sub-bandgap quasi-Fermi level splittings^12^.

4 $$\begin{aligned} J_\textrm{tot}&= J_\textrm{cv} + J_\textrm{ci} = J_\textrm{cv} + J_\textrm{iv} \,\,\, , \end{aligned}$$

5 $$\begin{aligned} qV&= \mu _\textrm{cv} = \mu _\textrm{ci} + \mu _\textrm{iv} \,\,\, . \end{aligned}$$

Finally, the total electrical output power density of the IB-TRD is determined by the product of the output voltage *V* and the corresponding total current density $J_\textrm{tot}$. The signs of *V* and $J_\textrm{tot}$ at the operating condition of TRD are opposite to those of a solar cell^5,6^. Here, $\mu _\textrm{ci}$ or $\mu _\textrm{iv}$ could have a positive value at the maximum power point while $\mu _\textrm{ci}$ + $\mu _\textrm{iv}$ has a negative value.

### Temperature dependence of bandgap energy and intrinsic carrier density

The temperature dependence of the bandgap energy was calculated using the Varshni equation^36^:

6 $$\begin{aligned} E_\textrm{g} (T) = E_\textrm{g} (0) - \frac{\alpha T^2}{T + \beta } \,\,\, , \end{aligned}$$

where $E_\textrm{g} (0)$ is the bandgap energy at *T* = 0 K, and $\alpha$ and $\beta$ are fitting parameters that describe characteristics of the considered material. The material parameters used in the calculation are summarized in Table 1.

**Table 1:** Material parameters of GaAs, Si, GaSb and Ge used for Eq. 6^37^.

Material	$E_\textrm{g}$(0) (eV)	$\alpha$ ($10^{-4}$ eV/K)	$\beta$ (K)
GaAs	1.517	5.5	225
Si	1.17 ^1^	4.73^1^	636^1^
GaSb	0.809	5.3	234
Ge	0.7412^2^	4.561^2^	210^2^

^1^ Ref. ^38^

^2^ Ref. ^36^

The intrinsic carrier density was calculated using the following equations, which were derived under the parabolic band approximation^39^:

7 $$\begin{aligned} n_{i} (T)&= \sqrt{N_\textrm{c} (T) N_\textrm{v} (T)} \exp \left\{ - \frac{E_\textrm{g}(T)}{2 k_\textrm{B} T} \right\} \,\,\, , \end{aligned}$$

8 $$\begin{aligned} N_\textrm{c} (T)&= 2 ( 2 \pi m_{dc}^{*} k_\textrm{B} T / h^2 )^{3/2} \,\,\, , \end{aligned}$$

9 $$\begin{aligned} N_\textrm{v} (T)&= 2 ( 2 \pi m_{dv}^{*} k_\textrm{B} T / h^2 )^{3/2} \,\, \, , \end{aligned}$$

10 $$\begin{aligned} m_{dc}^{*}&= M^{2/3} \left( {m_{t}^{*}}^2 m_{l}^{*} \right) ^{1/3} \,\,\, , \end{aligned}$$

11 $$\begin{aligned} m_{dv}^{*}&= \left\{ {m_\textrm{hh}^{*}}^{3/2} + {m_\textrm{lh}^{*}}^{3/2} + [m_\textrm{so}^{*} \exp (-\Delta / k_\textrm{B} T)]^{3/2} \right\} ^{2/3} \,\,\, , \end{aligned}$$

where $m_{dc}^{*}$ and $m_{dv}^{*}$ are the density-of-states effective masses in the CB and VB, respectively, *M* is the number of equivalent valleys at the points of the CB minimum, $m_{t}^{*}$ and $m_{l}^{*}$ are the transverse and longitudinal effective masses of the electron (associated with the ellipsoidal constant-energy surfaces), respectively, $m_\textrm{hh}^{*}$, $m_\textrm{lh}^{*}$ and $m_\textrm{so}^{*}$ are the effective masses of the heavy, light and spin–orbit split-off hole bands, respectively and $\Delta$ is the spin–orbit split-off energy. The material parameters used in the calculation are summarized in Table 2. It is known that Eq. 11 underestimates the hole effective mass for Si. Therefore, the intrinsic carrier density of Si was also calculated according to Refs.^39,40^, which takes the nonparabolicity in the VB into account.

**Table 2:** Material parameters of GaAs, Si, GaSb and Ge used for Eqs. 7–11^37^.

Material	$m_\textrm{e}^{*}$ ($m_{0}$)	$m_\textrm{hh}^{*}$ ($m_{0}$)	$m_\textrm{lh}^{*}$ ($m_{0}$)	$m_\textrm{so}^{*}$ ($m_{0}$)	$\Delta$ (meV)
GaAs	0.067	0.55	0.083	0.165	341
Si	$m_\textrm{tX}^{*}$: 0.1905 $m_\textrm{lX}^{*}$: 0.9163	0.528	0.157	0.29	42.62
GaSb	0.039	0.37	0.043	0.12	780
Ge	$m_\textrm{tL}^{*}$: 0.081 $m_\textrm{lL}^{*}$: 1.61	0.345	0.0427	0.095	295

## Results

### Current-density–voltage characteristics of an ideal IB-TRD

Figure 1**b** shows the current-density–chemical-potential characteristics of an ideal IB-TRD with $E_\textrm{cv}$ = 0.5 eV and $E_\textrm{ci}$ = 0.22 eV for $T_\textrm{c}$ = 500 K and $T_\textrm{e}$ = 300 K. Here, $J_\textrm{cv}$, $J_\textrm{ci}$ and $J_\textrm{iv}$ are the current densities caused by the three transition channels. It is noted that the horizontal axes for $J_\textrm{cv}$, $J_\textrm{ci}$ and $J_\textrm{iv}$ are the chemical potentials for the transitions with the bandgap energies of $E_\textrm{cv}$, $E_\textrm{ci}$ and $E_\textrm{iv}$ in the IB-TRD, respectively. Because the current density of a TRD significantly decreases as the bandgap energy is increased, the contribution of $J_\textrm{cv}$ to the total current density $J_\textrm{tot}$ in the IB-TRD is relatively small. Therefore, the current densities of $J_\textrm{ci}$ and $J_\textrm{iv}$ at the maximum power point plays a key role in achieving high output power densities. Figure 1c shows the current-density–voltage characteristics of the IB-TRD in Fig. 1b. The star in Fig. 1c represents the maximum power point ($\approx$ 34 W/m$^2$). All output power-density values provided in this work correspond to the value at the maximum power point.

### Dependence on the bandgap energy and the IB position

**Fig. 2:**
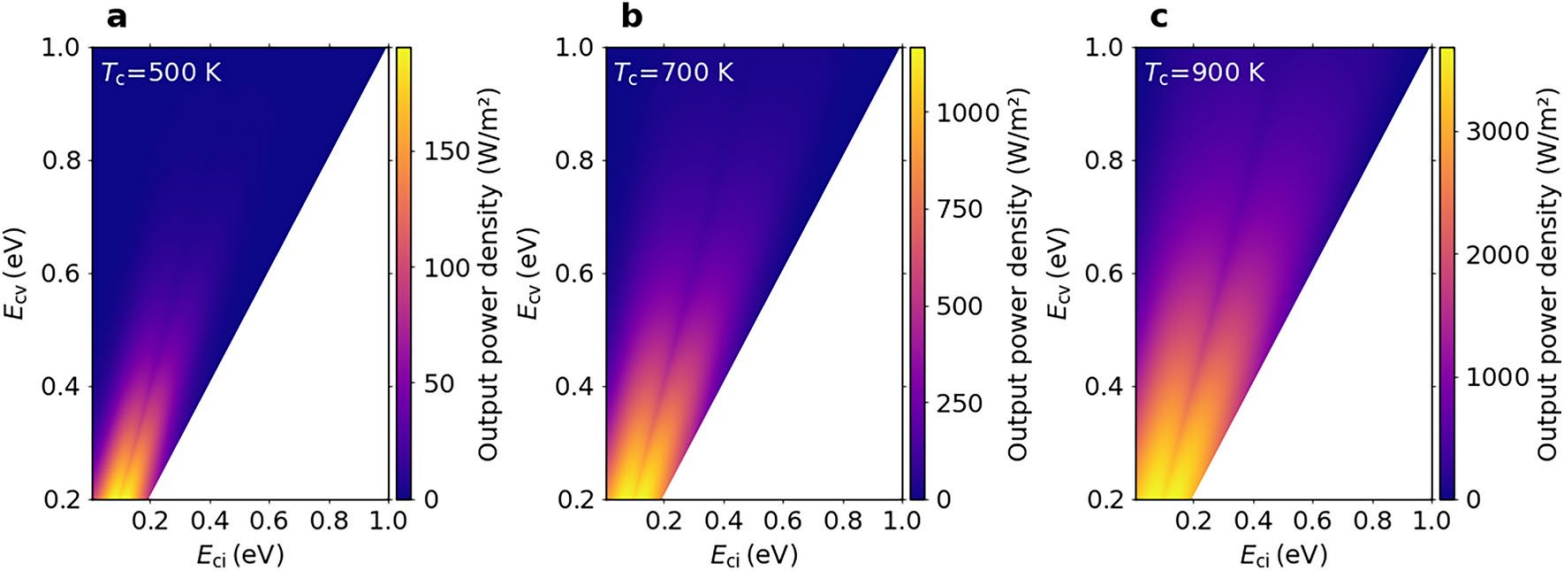
Two-dimensional colour maps of the output power density of an ideal IB-TRD. The output power density as functions of $E_\textrm{cv}$ and $E_\textrm{ci}$ for $T_\textrm{e}$ = 300 K and **a** $T_\textrm{c}$ = 500 K, **b** $T_\textrm{c}$ = 700 K and **c** $T_\textrm{c}$ = 900 K.

**Fig. 3:**
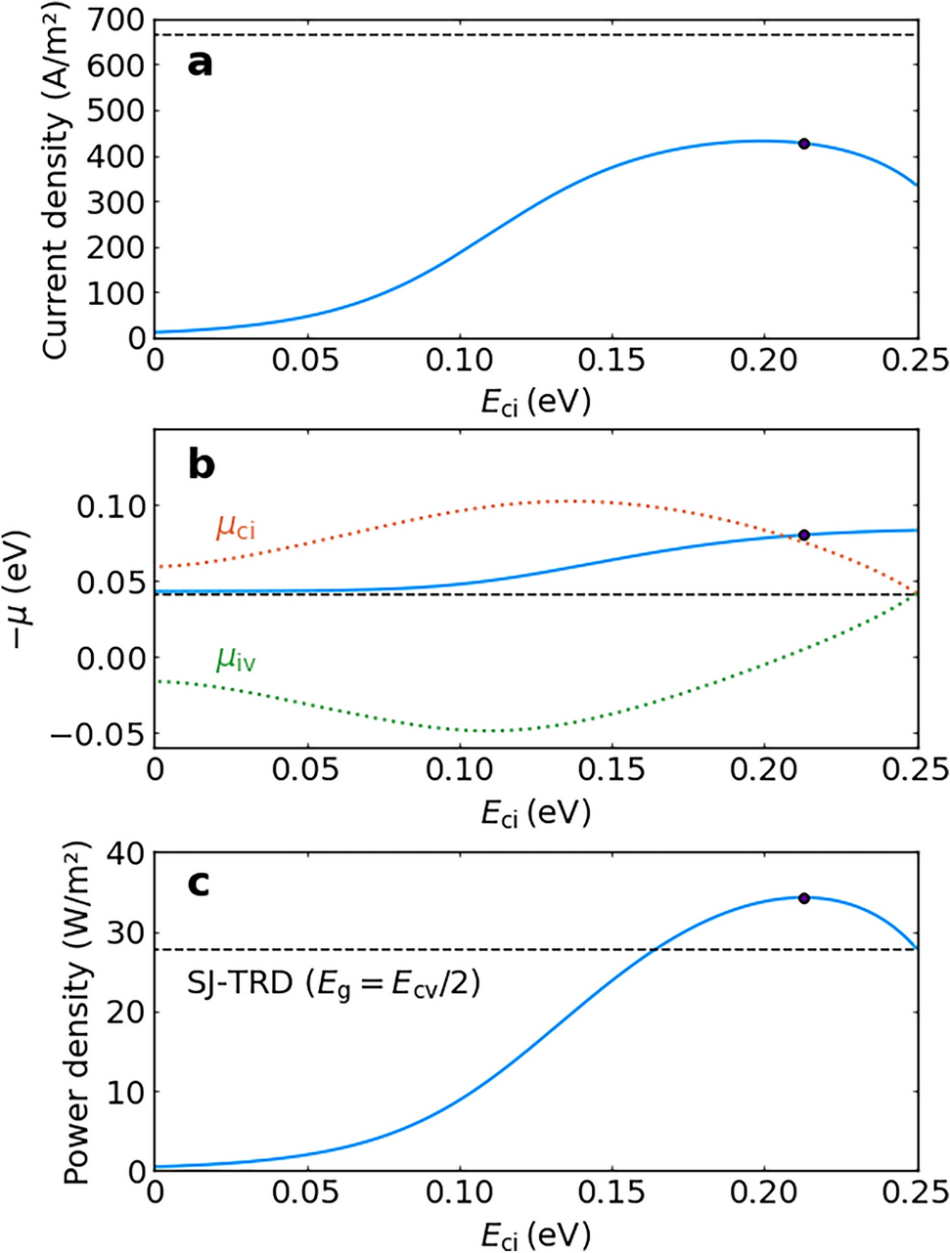
**IB position dependence of the current-density–voltage characteristics at the maximum power point.** The $E_\textrm{ci}$ dependencies of **a** the current density, **b** the chemical potential and **c** the output power density at the maximum power point for $E_\textrm{cv}$=0.5 eV at $T_\textrm{c}$=500 K and $T_\textrm{e}$=300 K. The red and green dotted curves in **b** represent $\mu _\textrm{ci}$ and $\mu _\textrm{iv}$, respectively. The circles indicate the optimal IB position. The horizontal dashed lines show the values of the SJ-TRD with the bandgap energy of $E_\textrm{cv}$/2.

Figure 2 shows the output power density of an ideal IB-TRD as functions of $E_\textrm{cv}$ and $E_\textrm{ci}$ for three different values of $T_\textrm{c}$ (500, 700 and 900 K) and $T_\textrm{e}$ = 300 K. The maximum output at $E_\textrm{cv}$ = 0.5 eV increases from $\approx$34 to $\approx$1904 W/m$^2$ as the temperature $T_\textrm{c}$ is increased from 500 to 900 K, because the current density increases with $T_\textrm{c}$. The short-circuit current density of an ideal IB-TRD with the IB in the middle of the bandgap becomes half the value that for an SJ-TRD with a bandgap equal to $E_\textrm{cv}$/2 when the contribution of $J_\textrm{cv}$ is negligible. On the other hand, we have discussed the output power density at the maximum power point. Figure 3 shows the $E_\textrm{ci}$ dependencies of the current-density–chemical-potential characteristics at the maximum power point for $E_\textrm{cv}$ = 0.5 eV at $T_\textrm{c}$ = 500 K and $T_\textrm{e}$ = 300 K. Figure 3a–c are the results of the current density, chemical potential and output power density, respectively. The red and green dotted curves in Fig. 3b represent $\mu _\textrm{ci}$ and $\mu _\textrm{iv}$, respectively. The circles indicate the values at the optimal IB position. The current density initially increases when the IB leaves the middle of $E_\textrm{cv}$, whereas the chemical potential $\mu _\textrm{cv} = \mu _\textrm{ci} + \mu _\textrm{iv}$ is almost constant, resulting from the current matching constraint for the transitions via the IB. Besides, the current density tends to reduce when the IB is further away from the middle of $E_\textrm{cv}$ and the $\mu _\textrm{iv}$ has positive values because the short-circuit current density for $J_\textrm{iv}$ drastically decreases by increasing $E_\textrm{iv}$. Consequently, the output power density has a maximum value when the IB is away from the middle of $E_\textrm{cv}$. Note that, in each figure in Fig. 2, the same maximum output value is achieved at two different values of $E_\textrm{ci}$ (hereafter referred to as the optimal IB positions, $E_\mathrm {ci, \, opt1}$ and $E_\mathrm {ci, \, opt2}$, where $E_\mathrm {ci, \, opt1} < E_\mathrm {ci, \, opt2}$) for each $E_\textrm{cv}$ because the same power density is obtained by interchanging the values of $E_\textrm{ci}$ and $E_\textrm{iv}$. On the other hand, the horizontal dashed lines in Fig. 3 show the values of the SJ-TRD with the bandgap energy of $E_\textrm{cv}$/2. Therefore, Fig. 3c demonstrates that the output power density of the IB-TRD is greater than that of the SJ-TRD due to the voltage increase caused by the two-step transition by taking into account the overlap of the absorption coefficients.

**Fig. 4:**
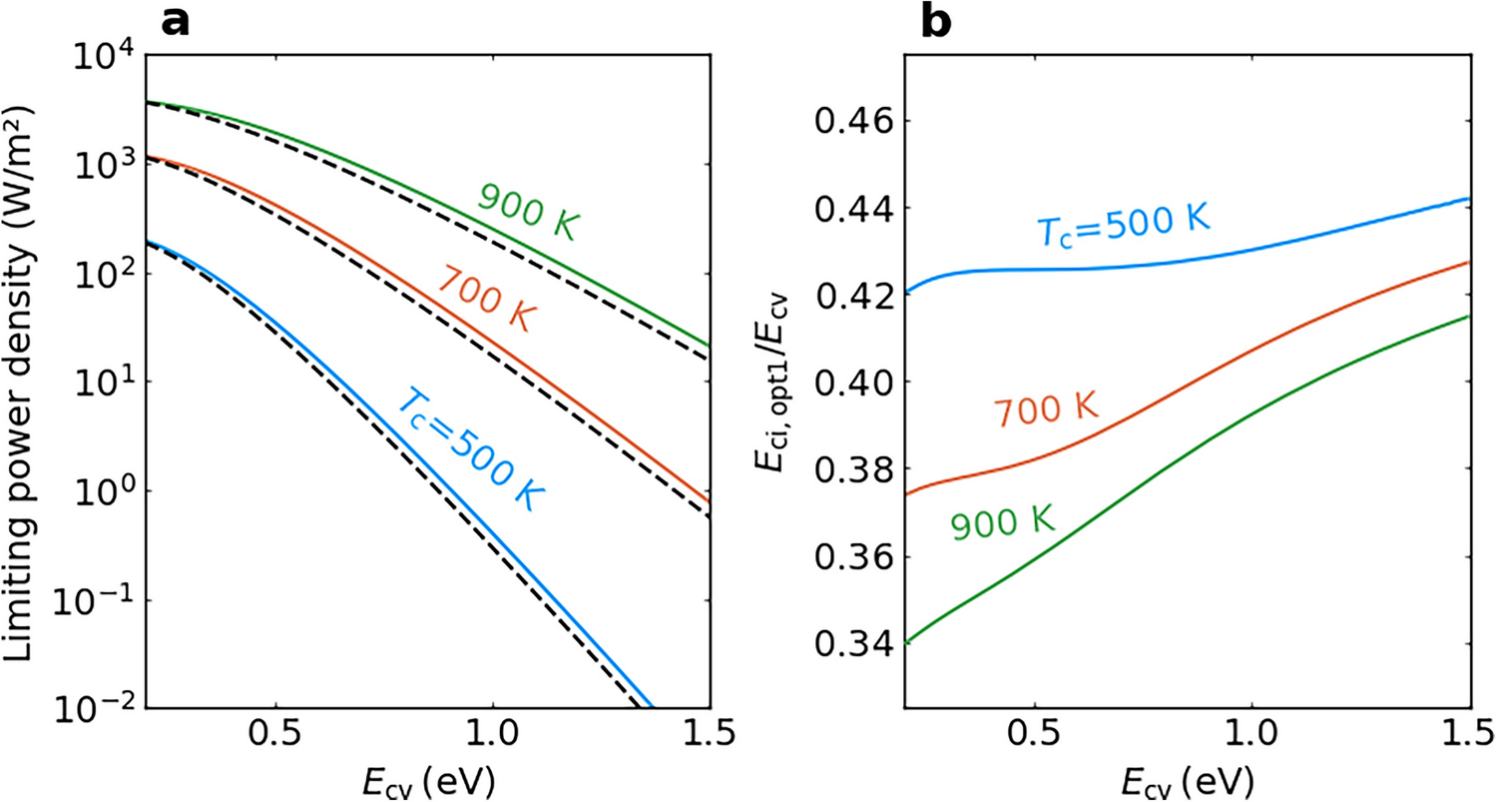
Bandgap energy dependence of the output power density of an ideal IB-TRD with an optimal IB position. **a** Output power density of an ideal IB-TRD as a function of $E_\textrm{cv}$ for $E_\textrm{ci} = E_\mathrm {ci, \, opt1}$ and $T_\textrm{c}$ = 500 K (blue solid curve), 700 K (orange solid curve) and 900 K (green solid curve). Black dashed curves indicate the output power density of an ideal SJ-TRD with a bandgap energy of $E_\textrm{cv}$/2. **b** $E_\mathrm {ci, \, opt1}$/$E_\textrm{cv}$ as a function of $E_\textrm{cv}$ for $T_\textrm{c}$ = 500 K (blue solid curve), 700 K (orange solid curve) and 900 K (green solid curve). The considered environmental temperature is 300 K.

Figure 4a shows the output power density of an ideal IB-TRD with an optimal IB position as a function of $E_\textrm{cv}$ for different device temperatures and $T_\textrm{e}$ = 300 K. The blue, orange and green solid curves indicate the results for $T_\textrm{c}$ = 500, 700 and 900 K, respectively, and the output power density is plotted on a logarithmic scale. The figure clarifies that reduction in the power density that is induced by an increase in $E_\textrm{cv}$ is smaller at higher $T_\textrm{c}$ values. The black dashed curves indicate the output power density of an ideal SJ-TRD with a bandgap energy of $E_\textrm{cv}$/2. The output power density of IB-TRD is greater than that of SJ-TRD at each $T_\textrm{c}$, which arises from the voltage boost effect uniquely appearing in IB-TRD. Figure 4b shows the value of $E_\mathrm {ci, \, opt1}$/$E_\textrm{cv}$ as a function of $E_\textrm{cv}$. The $E_\mathrm {ci, \, opt1}$ value increases almost linearly with $E_\textrm{cv}$ (with a slope of $\sim$1/2) and the three curves show that $E_\mathrm {ci, \, opt1}$ decreases as $T_\textrm{c}$ is increased. This behaviour is determined by the current matching constraint.

### Results for ideal IB-TRDs with bandgaps equal to GaAs, Si, GaSb and Ge as the host semiconductor

**Fig. 5:**
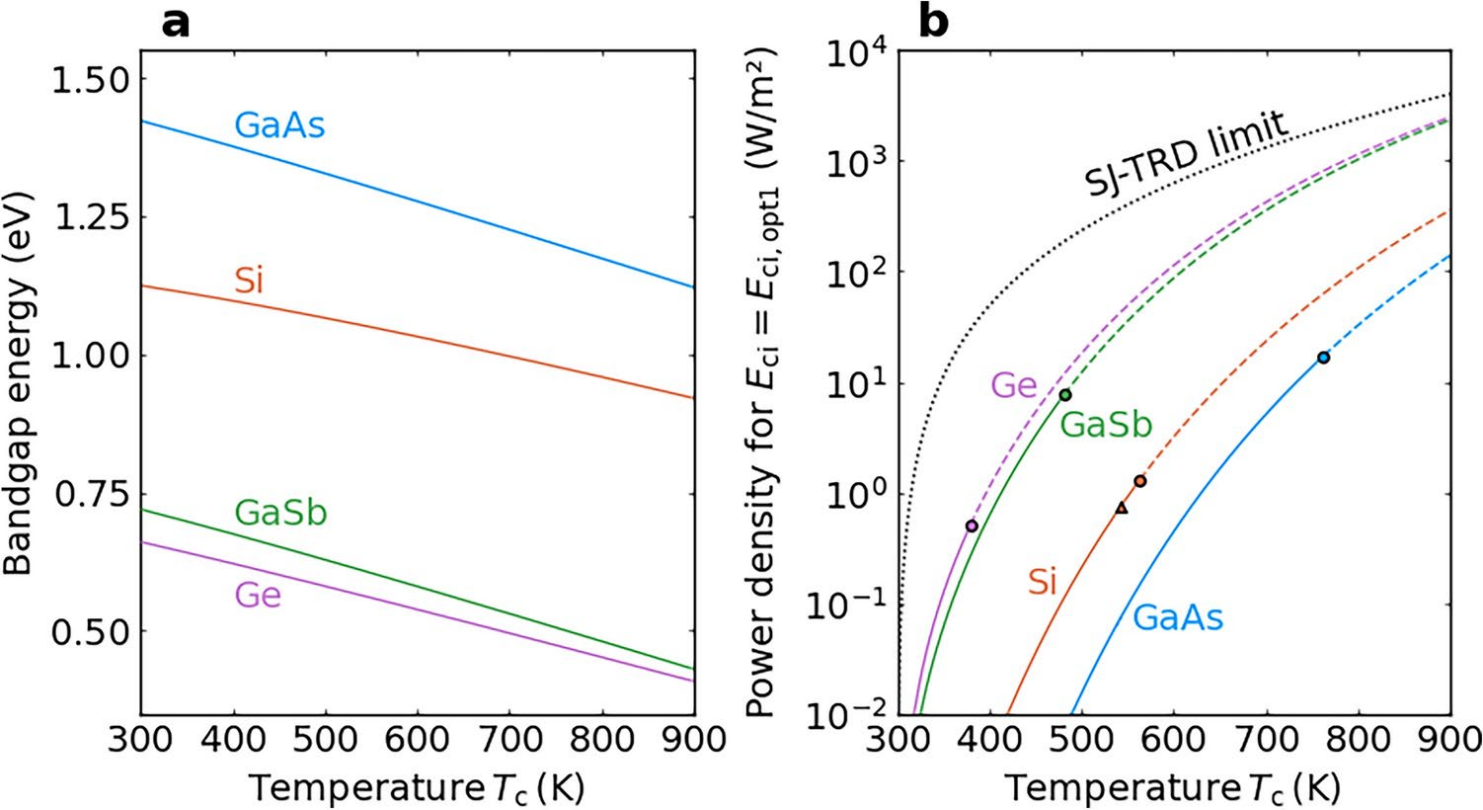
Comparison of ideal IB-TRDs that use different host semiconductors. **a** Temperature dependence of the bandgap energy for GaAs, Si, GaSb and Ge. **b** Device temperature dependence of the output power density of an ideal IB-TRD with an optimal IB position for different host semiconductors and $T_\textrm{e}$ = 300 K. The dotted line represents the output power-density limit of the SJ-TRD. The four circles indicate the power densities at temperatures where the predicted intrinsic carrier density is $10^{15}\mathrm{~cm}^{-3}$ (here, we used the parabolic band approximation). The solid and broken lines are used to emphasize the region with intrinsic carrier densities below $10^{15} \textrm{cm}^{-3}$. The triangle indicates the power density at the temperature where the intrinsic carrier density of Si is $10^{15} \textrm{cm}^{-3}$ when the nonparabolicity of the VB is taken into account.

Because the bandgap energy depends on the temperature, we have modeled the limit for ideal materials with bandgaps equal to GaAs, Si, GaSb and Ge. These semiconductors have also been considered in research on IB solar cells^13,14,19,41–43^. Figure 5a shows the temperature dependence of the bandgap energy for each of these four semiconductors. Note that these bandgap energies are more than one order of magnitude larger than the optimal bandgap energy for a SJ-TRD, $\approx$ 0.04 eV^5^ , in the whole temperature range in Fig. 5. Here, the optimal bandgap energy of the SJ-TRD changes from 0.045 to 0.034 eV as the temperature is increased. Because the bandgap energy decreases with increasing temperature, the temperature-induced increase in the output power density of a TRD can be attributed to two factors: an enhanced thermal emission due to a higher device temperature and an enhanced thermal emission due to a reduced bandgap energy.

Figure 5b shows the device temperature dependence of the output power density of an ideal IB-TRD with an optimal IB position for different host materials (GaAs, Si, GaSb and Ge) in the case of $T_\textrm{e}$ = 300 K. The output power density is plotted on a logarithmic scale. The dotted line represents the output power-density limit of the SJ-TRD. At $T_\textrm{c}$ = 500 K, the output power density of the Ge-based IB-TRD with an optimal IB position is one order of magnitude smaller than that of the SJ-TRD with the optimal bandgap energy of ≈ 0.04 eV, whereas the difference between the output power densities becomes smaller as $T_\textrm{c}$ is increased.

## Discussion

The practical absorptivities strongly depend on the temperature and the density of states of the IB. We clarified the temperature dependence of the IB filling by introducing the effective density of states. The electron density in the CB and IB, *n*, and the hole density in the VB and IB, *p*, at thermal equilibrium were calculated by the following equations:

12 $$\begin{aligned} n (T)&= N_\textrm{c} \exp \left( - \frac{E_\textrm{cv} - E_\textrm{F}}{k_\textrm{B}T} \right) + N_\textrm{IB} \frac{1}{1 + \exp \left( \frac{E_\textrm{iv} - E_\textrm{F}}{k_\textrm{B}T} \right) } \,\,\, , \end{aligned}$$

13 $$\begin{aligned} p (T)&= N_\textrm{v} \exp \left( - \frac{E_\textrm{F}}{k_\textrm{B}T} \right) + N_\textrm{IB} \left[ 1 - \frac{1}{1 + \exp \left( \frac{E_\textrm{iv} - E_\textrm{F}}{k_\textrm{B}T} \right) } \right] \,\,\, , \end{aligned}$$

where $N_\textrm{IB}$ is the effective density-of-states of the IB and $E_\textrm{F}$ is the Fermi level at thermal equilibrium. The first term indicates the electron (hole) density in the CB (VB) and the second term indicates the electron (hole) density in the IB. The Fermi-Dirac distribution is used in the second term because the Fermi level is close to the IB. The Fermi level at thermal equilibrium was calculated by satisfying $n = p$.

**Fig. 6:**
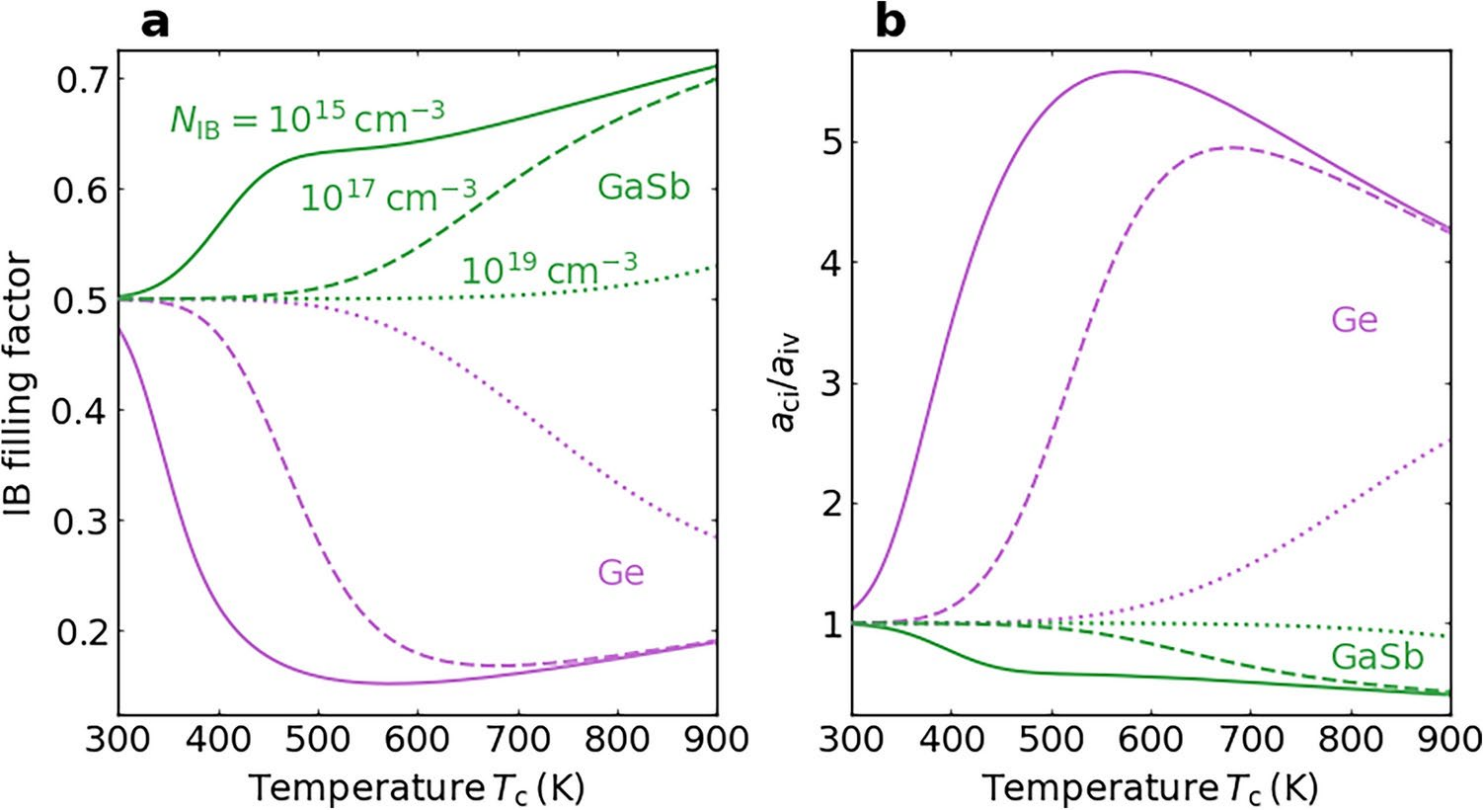
Temperature dependence of the IB filling factor and absorptivity ratio of an ideal IB-TRD with an optimal IB position. **a** IB filling factor and **b** absorptivity ratio of $a_\textrm{ci}$/$a_\textrm{iv}$ of an ideal IB-TRD, with the bandgap energies of Ge and GaSb, as a function of $T_\textrm{c}$. The IB is assumed to be located at the optimal position as well as in Fig. 5b. Solid, dashed and dotted curves indicate the results for the $N_\textrm{IB}$ of $10^{15}, 10^{17}$ and $10 ^{19} \mathrm{~cm}^{-3}$, respectively.

Here, we focus on the ideal IB-TRDs with bandgaps equal to Ge and GaSb because the optimal IB position is closer to the band edge of the host semiconductor compared to the IB-TRDs with bandgaps equal to Si and GaAs. Hence, the energy conversion of such IB-TRDs would significantly depend on temperature. Temperature dependence of the IB filling factor of an ideal IB-TRD is shown in Fig. 6a. The filling factor is zero for the fully depleted IB and unity for the IB filled with electrons. Here, we assumed that the IB is located at the optimal position for each temperature as well as in Fig. 5b. Solid, dashed and dotted curves indicate the results for the $N_\textrm{IB}$ of $10^{15}$, 10$^{17}$ and 10$^{19}\mathrm{~cm}^{-3}$, respectively. The IB filling factor is approximately 0.5 when the temperature is close to 300 K because the Fermi level is located close to the IB. On the other hand, the IB filling factor decreases with elevating the temperature for Ge, whereas that increases for GaSb. This different tendency results from the small effective mass of electrons in GaSb. The effective density-of-states of the electron, $N_\textrm{c}$, and that of the hole, $N_\textrm{v}$, have a value of the same order of magnitude in Ge. Since the optimal IB position leaves the middle of the bandgap with elevating the temperature as discussed in Fig. 4b, the Fermi level, and therefore the IB filling factor, decreases to satisfy $n = p$. On the other hand, the effective density-of-states of the electron is much smaller than that of the hole in GaSb. Therefore, the Fermi level increases with elevating the temperature to satisfy $n = p$ despite the optimal IB position leaving the middle of the bandgap. Besides, the IB filling factor remains close to 0.5 at higher temperatures with increasing the $N_\textrm{IB}$. Figure 6a indicates that the IB is not fully depleted or filled with electrons at high temperatures.

The ratio of the absorptivities for the transitions via the IB, $a_\textrm{ci}$/$a_\textrm{iv}$, was calculated using the IB filling factor. Here, we assumed that the absorptivity ratio is unity when the IB filling factor is 0.5. Figure 6b shows the temperature dependence of the absorptivity ratio of an ideal IB-TRD with the bandgap energies of Ge and GaSb. The absorptivity of $a_{ci}$ is larger than that of $a_\textrm{iv}$ for Ge and *vice versa* for GaSb.

**Fig. 7:**
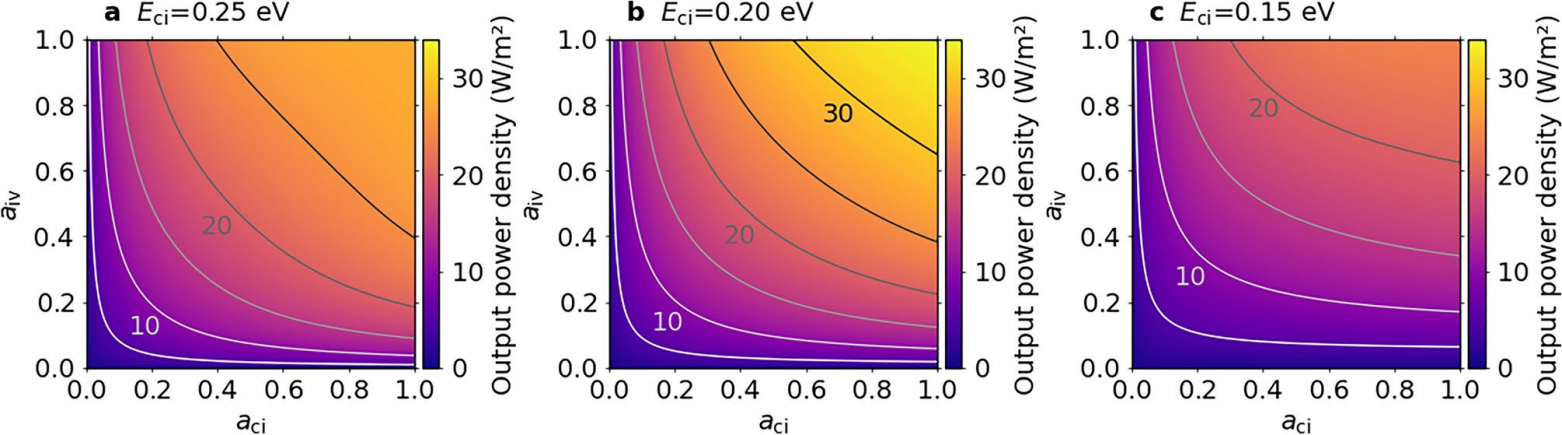
Two-dimensional colour maps of the output power density of an ideal IB-TRD taking into account the photon partitioning. Output power density as functions of $a_\textrm{ci}$ and $a_\textrm{iv}$ with $E_\textrm{cv}$ = 0.5 eV and **a** $E_\textrm{ci}$ = 0.25 eV, **b** $E_\textrm{ci}$ = 0.20 eV and **c** $E_\textrm{ci}$ = 0.15 eV, respectively, for $T_\textrm{c}$ = 500 K and $T_\textrm{e}$ = 300 K.

To clarify the effect of the partial absorptivity on the output power density, we calculated the output power density as functions of the absorptivities via the IB ($a_\textrm{ci}$ and $a_\textrm{iv}$), for example, with $E_\textrm{cv}$ = 0.5 eV and $E_\textrm{ci}$ = 0.25, 0.20 and 0.15 eV, for $T_\textrm{c}$ = 500 K and $T_\textrm{e}$ = 300 K in Fig. 7. When the IB appears at the middle of $E_\textrm{cv}$ (Fig. 7a), the maximum output power density is obtained when the two transitions share the photon flux equally. Conversely, when the IB shifts against the middle of $E_\textrm{cv}$, the results become asymmetric for $a_\textrm{ci}$ and $a_\textrm{iv}$. As shown in Fig. 7b and c, higher output power density is maintained when $a_\textrm{ci}$ is smaller than $a_\textrm{iv}$ because of the larger short-circuit current density of $J_\textrm{ci}$ than that of $J_\textrm{iv}$. According to the anisotropy between $a_\textrm{ci}$ and $a_\textrm{iv}$, photon partitioning needs to be optimized to attain the maximum power density. These results suggest that the calculated output power density shown in Figs. 2, 3, 4 and 5 without considering photon partitioning is slightly underestimated when the IB leaves the middle of $E_\textrm{cv}$. Besides, Fig. 6b suggest that the smaller effective mass of electrons in GaSb than that of Ge is preferable at the optimal IB position in Fig. 5b for achieving high output power density. The magnitude of the absorptivity could increase with the density of states of the IB, whereas the absorptivity ratio of $a_\textrm{ci}$/$a_\textrm{iv}$ approaches 1 as shown in Fig. 6b.

When we take into account the effect of the reabsorption process, Eq. 2 is modified as follows:

14 $$\begin{aligned} \frac{J_i}{q}&= N(E_i, \infty , T_\textrm{c}, \mu _i, a_i^{*}) - \sum _{j \ne i} N(E_i, \infty , T_\textrm{c}, \mu _i, a_i^{*} a_j^{*}) \nonumber \\&\hspace{1cm} + \sum _{j \ne i} N(E_j, \infty , T_\textrm{c}, \mu _j, a_i^{*} a_\textrm{j}^{*}) - N(E_i, \infty , T_\textrm{e}, 0, a_i^{*}) \,\,\, . \end{aligned}$$

**Fig. 8:**
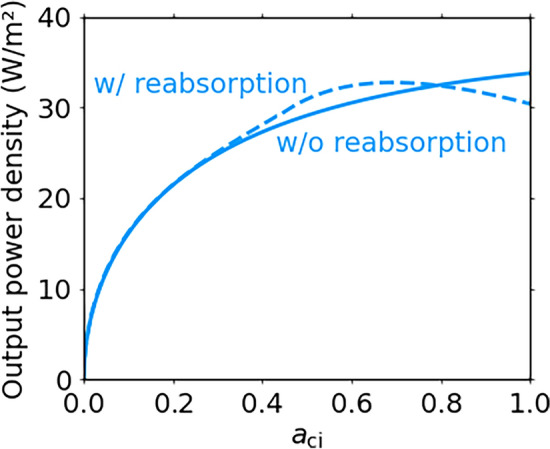
Effects of the reabsorption process on the output power density of the IB-TRD. The $a_\textrm{ci}$ dependence of the output power density for $E_\textrm{cv}$ = 0.5 eV, $E_\textrm{ci}$ = 0.2 eV and $a_\textrm{iv}$ = 1 at $T_\textrm{c}$ = 500 K and $T_\textrm{e}$ = 300 K. The solid and dashed curves represent the results without and with the reabsorption process.

The product of the absorptivities in the second and third terms describes the reabsorption process. The second term describes the reabsorption of photons emitted by the transition *i*, which mainly reduces $J_i$ for the transition with the narrower bandgap energy via the IB. Besides, the third term describes the reabsorption of photons emitted by the transition *j*, which mainly increases $J_i$ for the transition with the wider bandgap energy via the IB. Similar reabsorption process can be found for IB solar cells^30^ and tandem solar cells with the photon recycling process^33^. Figure 8 shows the effect of the reabsorption process on the output power density for $E_\textrm{cv}$ = 0.5 eV, $E_\textrm{ci}$ = 0.2 eV and $a_\textrm{iv}$ = 1 at $T_\textrm{c}$ = 500 K and $T_\textrm{e}$ = 300 K. The solid and dashed curves indicate the results without and with the reabsorption process, respectively. When photons are emitted by the larger sub-bandgap of $E_\textrm{iv}$, a part of the photons is reabsorbed by the smaller sub-bandgap of $E_\textrm{ci}$. This reabsorption process reduces the current density $J_\textrm{iv}$ and increases $J_\textrm{ci}$. Since $J_\textrm{iv}$ at $\mu _\textrm{iv}$ = 0 is smaller than $J_\textrm{ci}$ at $\mu _\textrm{ci}$ = 0 when $E_\textrm{iv}$ is larger than $E_\textrm{ci}$, the reabsorption process reduces the total current density $J_\textrm{tot}$ at the maximum power point due to the current matching constraint. The decreased $J_\textrm{tot}$ results in a smaller output power density by considering the reabsorption process at $a_\textrm{ci}$ = 1 in Fig. 8. The output power density initially increases with reducing the absorptivity $a_\textrm{ci}$ because the current density $J_\textrm{iv}$ increases when the reabsorbed photon density is reduced, and that tends to decrease when $a_\textrm{ci}$ further decreases. The output power density in Fig. 8 takes a maximum value at a certain point ($a_\textrm{ci}$ = $\sim$0.69). So far, optimal device thickness has been reported for the IB solar cells with an overlap in the absorption coefficients^30^. Figure 8 indicates that the reabsorption process modifies the optimal photon partitioning condition in the IB-TRDs as well as that in the IB solar cells. The maximum output power density slightly reduces by taking into account the reabsorption process, whereas that is still greater than that of the SJ-TRD with the bandgap energy of $E_\textrm{cv}$/2 as shown in Figs. 3c and 8.

Typical Si solar cells use base doping concentrations in the range from 1$\times 10^{15} \textrm{to }3 \times 10 ^{16}\mathrm{~cm}^{-3}$^44^ and the intrinsic carrier density needs to be significantly lower to form a *p–n* junction at the operating temperature. The four circles in Fig. 5b indicate the output power densities obtained using an optimal IB position and a temperature where the intrinsic carrier density is $10^{15}\mathrm{~cm}^{-3}$. Here, the intrinsic carrier density of $10^{15}\mathrm{~cm}^{-3}$ is considered as a guideline for the cutoff value changing the diode property. If we use the parabolic band approximation, we find that the intrinsic carrier densities of Ge, GaSb, Si and GaAs start to exceed $10^{15} \textrm{cm}^{-3}$ at $T_\textrm{c} \approx$ 380, 480, 560 and 760 K, respectively. Our calculated intrinsic carrier densities of Ge, GaSb and GaAs are consistent with the reported values^45,46^, but the value for Si is underestimated; when the nonparabolicity in the VB is taken into account, the intrinsic carrier density of Si starts to exceed $10^{15}\mathrm{~cm}^{-3}$ at $T_\textrm{c} \approx$ 540 K (the triangle in Fig. 5b)^39,40^. The output power densities of the ideal GaAs- and GaSb-based IB-TRDs with an optimal IB position and an intrinsic carrier density of $10^{15}\mathrm{~cm}^{-3}$ are larger than those of the ideal Si- and Ge-based IB-TRDs owing to the smaller electron effective masses of GaAs and GaSb. An output power density of $\approx$ 17 W/m$^2$ is estimated for the IB-TRD that uses GaAs with an intrinsic carrier density of $10^{15}\mathrm{~cm}^{-3}$ ($T_\textrm{c} \simeq$ 760 K).

So far, the high-temperature operation of IB-TRDs has been discussed (where a relatively high temperature of $T_\textrm{e}$ = 300 K can be used for the cold reservoir). On the other hand, the output power-density limit of an SJ-TRD operating at $T_\textrm{c}$ = 300 K is $\approx$ 55 W/m$^2$ if $T_\textrm{e}$ = 3 K^4^. Furthermore, if we consider the radiative limit and a bandgap energy of 0.013 eV, an SJ-TRD at $T_\textrm{c}$ = 300 K can generate $\approx$ 15 W/m$^2$ in the case of an atmospheric temperature of 270 K and a relative humidity of 40%^47^. However, the output power density of a SJ-TRD under the same atmospheric conditions is only $\approx$ 5.4 W/m$^2$ if we use a bandgap energy of 0.1 eV. We would like to emphasize that, under the same atmospheric conditions, the output power density of an IB-TRD with $E_\textrm{ci} = E_\textrm{cv}/2$ can reach $\approx$ 5.6 W/m$^2$ with a bandgap energy of 0.2 eV. Here, the optimal IB positions, $E_\mathrm {ci, \, opt1}$ and $E_\mathrm {ci, \, opt2}$, show a tendency to approach $E_\textrm{cv}/2$ with decreasing $E_\textrm{cv}$ when $E_\textrm{cv}$ is smaller than 0.5 eV.

It is noted that nonradiative processes drastically reduce the power densities of TRDs^5,6^; both the voltage and output power at the maximum power point decrease approximately linearly with the external luminescent efficiency as it is reduced in the SJ-TRD^6^. On the other hand, if the short-circuit current density of $J_\textrm{ci}$ is larger than that of $J_\textrm{iv}$ in the IB-TRD, the reduction of $V_\textrm{iv}$ at the maximum power point could have a small impact because the total voltage is mainly determined by $V_\textrm{ci}$.

As a final remark, we point out that an IB-TRD can also operate as a solar cell during daytime, because the bandgap energy $E_\textrm{cv}$ can be sufficiently large in such a device (compared to the bandgap energy of a SJ-TRD consisting of narrow-bandgap semiconductors). If we use the IB-TRDs based on GaAs, Si, GaSb and Ge as solar cells ($T_\textrm{c}$ = 300 K; AM1.5G^48^, 1 sun illumination with a total power density of 1000 W/m$^{2}$) and choose $E_\textrm{ci} = E_\textrm{cv}/2$, the output power densities in the radiative limit are approximately 274, 207, 92 and 75 W/m$^{2}$, respectively While these power densities are smaller than the corresponding values of the Shockley–Queisser limit for SJ solar cells^49^, the IB-TRD is potentially useful for applications that require operation under both positive and negative illumination conditions.

In conclusion, we have presented detailed-balance calculation results on the electrical output power density of an IB-TRD in the radiative limit. We showed that the output power density increases with $T_\textrm{c}$ and that $E_\textrm{ci, opt1}$ decreases as $T_\textrm{c}$ is increased, which is a result of the current matching constraint. Because the intrinsic carrier density needs to be significantly lower than the doping concentration to form a *p–n* junction at the operating temperature, IB-TRDs can be advantageous compared to SJ-TRDs consisting of narrow-bandgap semiconductors such as HgCdTe and InSb. An output power density of $\approx$ 17 W/m$^2$ was estimated for an IB-TRD with an optimal IB position that uses GaAs with an intrinsic carrier density of $10^{15}\mathrm{~cm}^{-3}$ ($T_\textrm{c} \simeq$ 760 K). Furthermore, in the case of a device temperature of 300 K, an atmospheric temperature of 270 K and a relative humidity of 40%, an IB-TRD with a bandgap energy of 0.2 eV has an output power density that is comparable to that of a SJ-TRD with a bandgap energy of 0.1 eV. IB-TRD devices may be considered for applications where operation under both positive and negative illumination conditions is required.

## Data Availability

The data that support the findings of this study are available from the corresponding author upon reasonable request.

## Code availability

The computer codes used in this study are available from the corresponding author upon reasonable request.
